# Advances in recombinant protein bioprocessing: integrating technological innovation and environmental sustainability

**DOI:** 10.1007/s10529-026-03750-4

**Published:** 2026-08-12

**Authors:** Milena de Freitas Trindade Mota, Luis Gustavo Carvalho Pacheco, Cesar Augusto Piedrahita Aguirre, Elaine Christine de Magalhães Cabral Albuquerque

**Affiliations:** Federal University of Bahia: Universidade Federal da Bahia, Salvador, Bahia Brazil

**Keywords:** Biotechnology, Bioengineering, *Escherichia coli*, Bioreactors, TRL and BRL, Sustainability

## Abstract

This study aims to provide an integrated assessment of recombinant protein bioprocessing by combining technological maturity frameworks (TRL/BRL) with environmental sustainability indicators. A structured literature-based analysis was conducted, covering recent advances in bioprocess engineering, including precision fermentation, artificial intelligence-assisted control, and sustainability assessment tools such as life cycle analysis (LCA) and process mass intensity (PMI). Recombinant protein production using microbial systems, such as *Escherichia coli,* demonstrates high scalability and cost-efficiency, with reported emissions significantly lower than those of conventional pharmaceutical synthesis (≈ 20 vs 351 kg CO_2_ eq/kg product). However, critical gaps persist between laboratory-scale innovation and industrial implementation, particularly in emerging economies. The integration of digital tools, including AI and digital twins, shows strong potential to improve process optimization and scale-up success rates. Bridging the gap between technological development and industrial deployment requires integrating process design, sustainability metrics, and readiness frameworks. Targeted innovation strategies are essential to accelerate biopharmaceutical development and enhance global health outcomes.

## Introduction

The efficient production of recombinant proteins is essential and considered one of the fundamental pillars of modern biotechnology. Significant contributions in this area have emerged from projects focused on genomes, melanoma screening, and enzyme optimization strategies, resulting in large quantities of recombinant proteins. In this context, the evaluation of expression systems has led to the development of small bioreactors for analyzing bioprocesses and their respective yields (Frey and Krull 2020).

The realization of large-scale production of recombinant proteins remains challenging. These challenges include posttranslational modifications, efficient resource bioconversion, and optimizing the bioprocess chain (upstream, bioproduction, and downstream processes). This requires further improvements and research to increase protein titers, space–time yields, and production rates. Nevertheless, recombinant proteins produced via precision fermentation hold great promise as functional biopharmaceuticals for clinical applications. Therefore, this study aims to provide an integrative evaluation of recombinant protein bioprocessing by combining technological, environmental, and process innovation perspectives, and to highlight key gaps between academic research and industrial implementation.

## Technological aspects

The generation of recombinant proteins represents a foundational aspect of contemporary biotechnology, facilitating the development of biopharmaceuticals, industrial enzymes, and molecules of therapeutic significance. Selecting an appropriate expression system is not just important; it is crucial for optimizing biopharmaceutical production (to ensure elevated yields) and enabling industrial scalability (Hemamalini et al. 2019).

Many systems are available for expressing recombinant proteins, as summarized in Table 1, microbial systems generally achieve higher volumetric titers compared to mammalian platforms. These values are representative and may vary significantly depending on the target protein and process conditions.

**Table 1:** Representative performance ranges of recombinant protein expression systems based on selected studies

System	Typical titer (mg/L)/productivity trend	Key advantages/key limitations
*Escherichia coli*	~ 50–400/High	Fast growth, low cost, high yield for simple proteins/No glycosylation, inclusion bodies
Yeast (*Pichia pastoris*)	~ 40–180/Moderate high	Secretion capability, partial PTMs/Hyperglycosylation, variability
Mammalian cells	~ 30–150/Low moderate	Human-like glycosylation, high product quality/High cost, low productivity

^*^Values based on representative comparative studies (Sarramegna et al. 2003; Hartwell et al. 2024)

Another approach used for recombinant protein expression is the mammalian host system, such as Chinese Hamster Ovary (CHO) cells. Since the first FDA approval of Activase®, over 100 recombinant proteins have utilized mammalian systems like CHO cells, primarily due to their ability to produce proteins with human-like glycosylation. However, disadvantages include pathogenic contaminants, slow growth, high production costs, associated with complex upstream cultivation and downstream processing, which is typically a major contributor to overall cost-of-goods.

The use of genetically modified microorganisms (specifically *E. col*i) for protein or peptide production via fermentation offers many advantages, including large-scale production, a well-studied system, and rapid cell growth. Advances in fermentation process techniques can enhance efficiency and sustainability. Precision fermentation has emerged as the most suitable tool in this process, which focuses on producing substances from abundant, low-cost substrates by targeted genetic modifications that reprogram microorganisms’ metabolic pathways. Precision fermentation integrates cutting-edge process engineering with a commitment to sustainable development.

## Innovations in bioprocesses and biopharmaceutical production

Recent advancements in bioprocess engineering for recombinant protein production, Table 2, have been largely driven by the pressing demand for more efficient, scalable, and cost-effective processes within the biopharmaceutical industry. In this context, precision fermentation emerges as a highly competitive and scalable platform for the production of high-value biomolecules, including specialized enzymes and therapeutic proteins.

**Table 2:** Technological trends in recombinant protein bioprocessing

Trend	Description	Advantages	Limitations	Industrial relevance
Precision Fermentation (Wan, et al., 2022)	The utilization of genetically modified microorganisms for the production of specific proteins, vitamins, and lipids	An environmentally sustainable alternative to traditional livestock farming, characterized by enhanced efficiency in substrate conversion and a reduced ecological footprint	Scale-up challenges, strain stability	Increasing adoption in industrial biotechnology
Process Systems Engineering (PSE) (Vinestock et al. 2024)	Optimization of fermentation processes using mathematical modeling and life cycle analysis	Reduced costs and environmental impact, better production planning	Model complexity, data dependency	Widely applied in process optimization
Advanced Process Control (Wan, et al., 2022)	Application of smart sensors and predictive control to optimize fermentation parameters	Greater productivity and yield, better control of the quality of the final product	High implementation cost	Standard in large-scale facilities
Artificial Intelligence in Fermentation (Xia et al. 2025)	Using machine learning for process modeling, production optimization, and fermentation control	Reduced development time, increased production efficiency, and better performance prediction	Data availability, model generalization issues	Emerging tool with increasing industrial interest
Use of Alternative Substrates (Philippini et al. 2020)	Exploitation of agricultural residues and industrial by-products as carbon sources	Reduced raw material costs, lower environmental impact	Variability in composition, supply chain issues	Growing interest in circular bioeconomy
Hybrid Modeling and Digital Twins (Shah et al. 2023)	A combination of physics-based models and artificial intelligence for simulation and optimization of fermentation processes	Improved process predictability and better adaptation to different operating conditions	High computational demand, validation challenges	Promising for industrial scale-up
Reinforcement Learning-Based Control Methods (Nagabhushanamgari Rajasekhar et al. 2024)	Using reinforcement learning algorithms to optimize fermentation processes in real time	Automatic adjustment of operational variables, increased efficiency, and process automation	Limited real-world validation	Early-stage development
Environmental Impact and Sustainability Analysis (Fröhling and Hiete 2020)	Integration of Life Cycle Assessment (LCA) into decision-making on fermentation processes	Development of more sustainable and economically viable processes	Data-intensive, complex implementation	Increasingly required in industrial design

Unlike traditional fermentation, which often yields a mix of products, precision fermentation offers highly controlled, high-yield production of target compounds. Recent advances in bioprocess control have integrated smart sensors, computational modeling, and machine learning to enhance process efficiency and precision. Smart sensors enable real-time monitoring of key parameters, such as pH, temperature, and dissolved oxygen, in bioreactors. Computational models can simulate cellular growth dynamics during fermentation, while machine learning algorithms are increasingly used to predict optimal operating conditions to maximize productivity. AI, specifically digital twins, can facilitate the scale-up from lab to industrial bioreactors. These strategies are fundamental to improving the productivity and sustainability of fermentation processes (Vinestock et al. 2024). The primary technological trends are precision fermentation, process systems engineering, and advanced process control.

Recent studies have demonstrated the practical application of artificial intelligence in bioprocess optimization. For example, machine learning models have been successfully applied to predict optimal feeding strategies in fed-batch fermentation, thereby improving biomass accumulation and product yield. Similarly, digital twin models have been implemented to simulate large-scale bioreactor conditions, enabling more accurate scale-up by reproducing gradients in oxygen transfer and nutrient distribution observed in industrial systems.(Richter et al. 2025).

## Technological maturity assessment: TRL and BRL

The technological maturity assessment is essential for consolidating innovations in biotechnology, particularly in the production of biopharmaceuticals and recombinant vaccines. The Technology Readiness Levels (TRL) scale is widely used to quantify the development stage of technologies, ranging from theoretical conception to industrial validation (Mankins 1995). It comprises nine levels grouped into three stages: proof of concept (TRL 1–3), laboratory validation (TRL 4–6), and commercial implementation (TRL 7–9).

A critical phase within this framework is the “valley of death,” a stage where many promising technologies fail due to funding gaps, technical barriers, or market uncertainty. This phase is crucial for validating technological feasibility and scalability. Studies indicate that successful progression requires both proof of concept and competitive scalability (Velho et al. 2018). Notably, only one in 5000–10,000 innovations reaches commercialization (Ding and Eliashberg 2002), highlighting the importance of strategies that facilitate technological translation.

This challenge is particularly relevant in biotechnological processes, especially in pharmaceutical development and the development of therapeutic devices to achieve commercial viability. Despite advances in upstream fermentation strategies, downstream processing remains a significant cost barrier in recombinant protein production, accounting for up to 80% of total process costs. The choice of purification method depends on the protein’s characteristics (e.g., molecular weight, solubility, pI) and the production scale. The laboratory-scale method prioritizes high purity, whereas the industrial-scale method focuses on maximum recovery, efficiency, and cost reduction. The biotechnology sector plays a central role in technology transfer among academia, industry, and government (Schoonmaker et al. 2013; Kampers et al. 2022), underscoring its importance to innovation ecosystems.

Given TRL’s limitations in capturing the specificities of biopharmaceutical manufacturing, complementary frameworks have been proposed. The Biomanufacturing Readiness Levels (BRL), developed by the National Institute for Innovation in Manufacturing Biopharmaceuticals (NIIMBL), provide a more tailored assessment of readiness for clinical and industrial production. Similarly, the Business Readiness Level (BRL) framework addresses commercialization aspects, structured into three phases: concept development (BRL 1–3), proof of concept (BRL 4–7), and concept realization (BRL 8–9) (Kedia et al. 2022).

Patent analysis also contributes to maturity assessment by identifying technological trends and innovation gaps. Intermediate TRLs can be inferred from scientific publications (TRL 3), academic patents (TRL 4), and national patents (TRL 5–6) (Nesta and Patel 2006; Quintella et al. 2023). While scientific output (TRL 3) remains robust in sectors like biopharmaceuticals, a significant gap persists in translating these findings into applied technologies (TRL 4–5), hindering large-scale industrial implementation (Florêncio et al. 2020; Quintella et al. 2024).

However, TRL alone does not capture economic feasibility, which is critical for industrial implementation. A thorough evaluation of economic metrics, including cost of goods sold, capital and operating expenditures, and projected return on investment, alongside a comprehensive commercial valuation, is essential to establishing the feasibility and market competitiveness of recombinant protein production processes. So, a qualitative techno-economic assessment (TEA) perspective is important and enhances the evaluation of biotechnological processes. Key economic drivers, including capital expenditure (CAPEX), operational expenditure (OPEX), and cost of goods (COGs), directly influence scalability and commercial viability (Farid 2007; Strube et al. 2018). CAPEX is mainly associated with facility design and equipment, while OPEX is driven by raw materials, labor, utilities, and consumables. Process intensification strategies, particularly the transition from batch to continuous manufacturing, have demonstrated significant economic advantages, including reductions in COGs due to improved productivity, smaller facility footprint, and more efficient resource utilization (Pollock et al. 2017). Continuous bioprocessing has been associated with significant reductions in cost of goods, with reported decreases of approximately 73–83% for microbial systems and 35–68% for monoclonal antibody production, depending on scale and process configuration (Gupta et al. 2019).

Moreover, although industrial secrecy and patent protection can limit knowledge dissemination, emerging open biomanufacturing initiatives and pre-competitive consortia are helping to overcome these barriers. Organizations such as the National Institute for Innovation in Manufacturing Biopharmaceuticals (NIIMBL) and the Advanced Mammalian Biomanufacturing Innovation Center (AMBIC), along with networks, promote collaborative data sharing, process standardization, and open-access frameworks (“Big Data Program—NIIMBL”, 2026; *What Is AMBIC?—*AMBIC 2020). These initiatives facilitate the exchange of non-proprietary knowledge, reduce duplication of efforts, and accelerate innovation, particularly in overcoming bottlenecks associated with the “valley of death.”

Finally, the production of recombinant proteins and biopharmaceuticals depends on advances in bioprocess engineering (upstream, downstream, optimization) and on overcoming regulatory and scale-up challenges. The integration of TRL, BRL, and TEA approaches provides a robust and multidimensional framework for assessing technological maturity, supporting the advancement of biotechnology as a strategic sector for innovation and industrial development.

## Environmental aspect

Several assessment methods, such as the E-factor, PMI, and LCA, are used to measure the environmental impacts of biopharmaceutical production (Wowra et al. 2023). Although more robust and standardized, LCA is often overlooked due to its complexity, significant time investment, and reliance on data typically unavailable during early-stage development (Wowra et al. 2023). Consequently, there is a lack of systematic integration of environmental sustainability assessment into the early stages of bioprocess development, and the use of techno-economic (TEA) and life-cycle (LCA) assessment tools during these initial phases (Carruthers and Lee 2022). This gap hinders the translation of laboratory innovations into commercially viable and truly sustainable biomanufacturing platforms. As a result, there has been increasing discussion on the importance of Life Cycle Assessment (LCA) across all stages of biopharmaceutical production.

## Environmental impacts of bioprocesses

Bioprocesses face sustainability challenges linked to high-emission inputs; intensive agricultural sectors, particularly enteric fermentation,contribute significantly to global greenhouse gas inventories and the environmental footprint of culture media (Brazil 2022). This reinforces the need to assess the origin of animal- and agro-industrial-derived substrates in bioprocess culture media, whose demand sustains emission-intensive systems. A granular analysis of inputs, carbon and nitrogen sources, vitamins, water, across their supply chains is essential; however, comprehensive assessments tracing the environmental impacts of all inputs used in biopharmaceutical production remain scarce (Chatzipanagiotou et al. 2025).

Furthermore, the downstream process in biopharmaceutical production is a significant water consumer, accounting for up to 95% of all components used in this stage (Lorek et al. 2025). This water is primarily used for equipment cleaning and buffer preparation, both of which are high-volume processes. However, the current high water levels and buffer consumption directly conflict with global commitments set out in the Sustainable Development Goals. With the projected growth in demand in the biopharmaceutical market, developing and implementing more sustainable strategies is crucial. These strategies are necessary to minimize the environmental impacts of these processes and ensure the industry’s alignment with the Sustainable Development Goals (“The 17 Goals” n.d.).

Another critical environmental challenge is managing waste generated during bioprocesses. Traditionally, the biopharmaceutical industry focuses on product purity, productivity, and yield, with sustainable waste management often taking a back seat. However, strategies such as the promising transition from batch to continuous operations have shown potential in reducing water and buffer consumption, thereby enhancing process efficiency.

## Innovations for sustainability

To mitigate environmental impacts, the biopharmaceutical industry has invested in continuous cell cultures and single-use technologies (SUTs). The SUTs eliminate CIP and SIP steps, seemingly reducing water and energy use compared to stainless steel (Eudralex 2015). However, Budzinski et al. (2022) reveal a misconception: cleanroom infrastructure accounts for 80% of the climate impact, while plastic waste contributes < 8%. Ding et al. (2022) add that a continuous SUT platform still consumes 4865 kg of water/kg product. Moreover, the E‑factor metric (excluding energy and CO_2_) masks electricity‑related burdens. For context, fermentation‑based products, such as recombinant proteins, emit 20 kg CO_2_ eq/kg, compared with 351 kg CO_2_ eq/kg for chemical synthesis (Etit et al. 2024).

Water-minimized downstream and buffer recycling directly affect LCA outcomes. For monoclonal antibody (mAb) primary recovery, single‑use filtration reduces cost of goods by up to 10% at a small scale (50 kg/year) compared to centrifugation, while also lowering water use and Processes Mass Intensity (PMI); at large scale (1000 kg/year), centrifugation is cheaper despite higher PMI—a trade‑off risk (Cataldo et al. 2020). For buffer recycling, risk‑aware assessment limits savings to 14–23% due to contamination risks (Satzer 2024), yet experimental CIP buffer recycling achieved 29% savings without compromising purity or yield (Lorek et al. 2025). Linking PMI to LCA indicators guides early design.

Thus, the perceived environmental benefit of SUTs is a contradiction rooted in the choice of metrics. Effective mitigation lies in renewable energy (up to 89% reduction) and process intensification, not in plastic recycling (which provides only 2% climate benefit) (Budzinski et al. 2022). Energy decarbonization, not waste management, should be the industry’s primary sustainability focus.

## Conclusion

The application of biotechnology to the production of recombinant proteins is a field of increasing relevance to the pharmaceutical and biomedical industry. Indeed, it has enabled substantial advances in the development of immunotherapies and biopharmaceuticals. Nevertheless, this process’s scalability and economic viability continue to present significant challenges, necessitating advanced optimization strategies and technological innovation. This study emphasizes that judicious selection of expression systems, the formulation of efficient purification protocols, and the integration of emerging technologies, such as artificial intelligence and continuous bioprocesses, are imperative for optimizing productivity and reducing operating costs. From an environmental standpoint, implementing sustainable practices can mitigate negative impacts and reduce the biopharmaceutical industry’s carbon footprint.

Therefore, this study highlights that the successful translation of recombinant protein technologies from the laboratory to industry depends on integrating technological maturity, economic feasibility, and environmental sustainability. While microbial systems offer clear advantages in scalability and cost, challenges related to downstream processing and regulatory requirements remain significant barriers. The combined use of TRL/BRL, TEA, and LCA frameworks provides a comprehensive approach to guide innovation and improve decision-making in bioprocess development.

Moreover, data availability is subject to industrial secrecy and patents, which hinder research.

## Data Availability

Data sharing is not applicable to this article as no new data were created or analyzed in this study.

## Abbreviations

TRL: Technology readiness levels
BRL: Biomanufacturing readiness levels
DNA: Deoxyribonucleic acid
CIP: Clean-in-place
SIP: Sterilize-in-place
AIT: Allergen immunotherapy
LCA: Life cycle assessment
SDGs: Sustainable development goals
NIIMBL: Innovation in manufacturing biopharmaceuticals
E-factor: Environmental factor
PMI: Process mass intensity
TEA: Techno economic analysis
